# Metabolic Syndrome, Kidney-Related Adiposity, and Kidney Microcirculation: Unraveling the Damage

**DOI:** 10.3390/biomedicines12122706

**Published:** 2024-11-27

**Authors:** Kyu Won Jang, Jin Hur, Dong Won Lee, Seo Rin Kim

**Affiliations:** 1 Division of Nephrology and Research Institute for Convergence of Biomedical Science and Technology, Pusan National University Yangsan Hospital, Yangsan 50612, Republic of Korea; rbdnjs0214@naver.com (K.W.J.); gene44@pusan.ac.kr (J.H.); dongwonlee@pusan.ac.kr (D.W.L.); 2 Department of Convergence Medicine, Pusan National University School of Medicine, Yangsan 50612, Republic of Korea; 3 Department of Internal Medicine, Pusan National University School of Medicine, Yangsan 50612, Republic of Korea

**Keywords:** metabolic syndrome, kidney microcirculation, visceral adiposity, kidney-related fat

## Abstract

Metabolic syndrome (MetS) is a cluster of interrelated risk factors, including insulin resistance, hypertension, dyslipidemia, and visceral adiposity, all of which contribute to kidney microvascular injury and the progression of chronic kidney disease (CKD). However, the specific impact of each component of MetS on kidney microcirculation remains unclear. Given the increasing prevalence of obesity, understanding how visceral fat—particularly fat surrounding the kidneys—affects kidney microcirculation is critical. This review examines the consequences of visceral obesity and other components of MetS on renal microcirculation. These kidney-related fat deposits can contribute to the mechanical compression of renal vasculature, promote inflammation and oxidative stress, and induce endothelial dysfunction, all of which accelerate kidney damage. Each factor of MetS initiates a series of hemodynamic and metabolic disturbances that impair kidney microcirculation, leading to vascular remodeling and microvascular rarefaction. The review concludes by discussing therapeutic strategies targeting the individual components of MetS, which have shown promise in alleviating inflammation and oxidative stress. Integrated approaches that address both of the components of MetS and kidney-related adiposity may improve renal outcomes and slow the progression of CKD.

## 1. Introduction

Metabolic syndrome (MetS), initially termed as ‘syndrome X’ or ‘insulin resistance syndrome’ by Reaven in 1988, encompasses a cluster of metabolic abnormalities, including insulin resistance, impaired glucose tolerance, elevated triglyceride (TG), low high-density lipoprotein (HDL) cholesterol levels, elevated blood pressure, and abdominal obesity [1]. The definition of MetS in adults has been established by several organizations, including the World Health Organization, the National Cholesterol Education Program, the International Diabetes Federation (IDF), and the National Heart, Lung, and Blood Institute. To harmonize these varied definitions, a joint task force outlined five diagnostic criteria for adult MetS, requiring the presence of at least three: (1) increased waist circumference (WC) according to population- and country-specific definitions; (2) systolic blood pressure ≥ 130 mmHg and/or diastolic blood pressure ≥ 85 mmHg or current treatment for hypertension; (3) fasting blood glucose ≥ 100 mg/dL or treatment for hyperglycemia; (4) TG ≥ 150 mg/dL or current treatment for elevated TG; and (5) HDL cholesterol < 40 mg/dL in men or <50 mg/dL in women, or treatment for reduced HDL cholesterol [2]. An increasing number of children and adolescents are affected by MetS due to rising obesity rates. Childhood MetS, which often persists into adulthood, differs from adult MetS in certain parameters but leads to similar complications, including an even greater cardiovascular risk. To address age-related differences, the IDF has defined MetS criteria for three age groups: 6 to <10 years, 10 to <16 years, and ≥16 years. Abdominal obesity (≥90th percentile) is a key criterion across all groups. Below age 10, MetS is not diagnosed, though weight reduction is emphasized. For ages 10 to <16, diagnosis requires abdominal obesity plus two of the following: TG ≥ 150 mg/dL, HDL-C < 40 mg/dL, systolic blood pressure ≥ 130 mmHg or diastolic blood pressure ≥ 85 mmHg, and fasting plasma glucose ≥ 100 mg/dL or previously diagnosed type 2 diabetes. For those ≥16, IDF adult criteria apply [3,4].

Obesity, defined by excessive body fat, is often estimated by body mass index (BMI) or body surface area; however, given the heterogeneity of obesity, individuals with the same BMI may have distinct body fat distribution and metabolic profiles [5]. Visceral adipose tissue is more metabolically active and harmful than subcutaneous fat. Visceral adiposity promotes the release of pro-inflammatory molecules, alterations in adipokine secretion, and elevated levels of free fatty acids, accompanied by insulin resistance [6]. Due to these cumulative effects, several studies have shown that visceral adipose tissue is associated with key components of MetS, such as type 2 diabetes, hypertension, obesity, and dyslipidemia, independently of BMI [7,8]. Among the visceral fat deposits, kidney-related adiposity—such as pararenal, perirenal, renal sinus, and parenchymal fat—that encases the kidney and provides mechanical support, may have detrimental effects on kidney health when present in excess. Recent studies have suggested a link between perirenal adipose tissue and kidney disease, indicating that kidney-related fat may play a role in both metabolic and kidney dysfunction [9,10]. Furthermore, individuals with increased renal sinus fat (RSF) were at a greater risk for developing chronic kidney disease (CKD), which persisted after adjustment for BMI or visceral adipose tissue [11].

MetS and obesity are significant contributors to the onset and progression of CKD, as well as to an increased risk of developing acute kidney injury [12]. Although the pathogenesis of CKD in individuals with overweight and obesity remains unclear, kidney damage in this population may present as obesity-related glomerulopathy, characterized by glomerulomegaly and focal segmental glomerulosclerosis [13]. Vascular damage is also a common feature in conditions such as diabetes, obesity, and MetS. Damage to the kidney’s microvasculature may play a crucial role in the progression of kidney disease. However, the impact of MetS on kidney microcirculation is still poorly understood. This review aims to explore the pathophysiology of MetS affecting kidney function, with a specific focus on kidney microcirculation and kidney-related adiposity. Additionally, it outlines strategies for the treatment of kidney diseases associated with MetS.

## 2. The Impact of Each Metabolic Syndrome Component on Kidney Microcirculation and Their Synergistic Interactions

### 2.1. Kidney Microcirculation

The kidney microvasculature’s complex structure supports its specialized physiological function. It consists of two distinct capillary networks: the glomerular and peritubular capillaries [14]. This microvasculature plays a crucial role in blood filtration, sodium excretion, and maintaining autoregulation through a combination of vascular and tubular mechanisms. These include the myogenic response and macula densa tubuloglomerular feedback, which help protect against hypertensive barotrauma [15,16]. The structure of glomerular capillaries regulates the distribution of blood flow and pressure, maintaining a balanced flow to conduit vessels. However, defects in the myogenic response and renal blood flow autoregulation can lead to increased glomerular pressure [17], capillary damage, and podocyte loss, resulting in hyperfiltration and a progressive decline in glomerular filtration rate (GFR) [18]. Peritubular capillaries deliver oxygen and nutrients to the tubular and interstitial cells in the kidney cortex. Because peritubular capillaries surround the renal tubules, the kidneys may be more susceptible to ischemic damage [14]. Defects in perfusion, even with normal renal blood flow, can lead to renal hypoxia, correlating with tubular injury [19].

### 2.2. Insulin Resistance and Impaired Glucose Tolerance

The impact of insulin resistance on renal microcirculation in patients with MetS arises from insulin’s role as a key regulator of factors such as nitric oxide (NO), endothelin-1 (ET-1), angiotensin II (AngII), and vascular endothelial growth factor (VEGF), all of which are critical for blood vessel dilation, constriction, and formation. In white adipose tissue, insulin activates endothelial NO synthase and increases NO through the phosphatidylinositol 3-kinase (PI3K)/protein kinase B (AKT) pathway and the mitogen-activated protein kinase (MAPK)/protein phosphatase-1 pathway [20]. Acute hyperinsulinemia with euglycemia has been shown to have a vasodilatory effect, linked to endothelium-derived NO, in vascular beds such as skeletal muscles in humans [21]. However, increased insulin levels in the kidneys lead to reduced NO synthesis, leading to vasoconstriction and an anti-natriuretic effect [22,23].

Insulin has also been shown to be associated with enhanced expression and circulating levels of ET-1, known as the vasoconstrictor of the renal vasculature [24]. In an obese rat model, an elevated expression of ET-1 in adipocytes via protein kinase C/MAPK pathways affects insulin release and reduces insulin sensitivity through the activation of ET type B receptors [25,26]. Studies with ET receptor antagonists, such as atrasentan and bosentan, have shown reduced circulating non-esterified free fatty acids and TG in high-fat diet mice [27]. Additionally, ET receptor antagonists play a role in reducing the gene expression of *transforming growth factor Β1 (TGFΒ1)* as well as type I, type III, and type IV collagens in diabetic glomeruli [28]. In the renal artery, elevated ET-1 activates the nuclear factor kappa B (NF-κB) signaling pathway, contributing to renal artery aging and kidney fibrosis, even in the absence of hypertension [29].

Hyperinsulinemia is also implicated in hypertension. Insulin reduces the expression of the kidney dopamine D1 receptor and its coupling to G-protein, inhibiting sodium-potassium (Na-K) ATPase and reducing sodium excretion [30]. Moreover, insulin regulates the expression of angII type 1 (AT1) and angII type 2 (AT2) receptors in the mesangial cells of obese rats [31,32]. In vitro studies indicate that hyperinsulinemia upregulates both AT1 and AT2 receptors in the kidney. AngII, through AT1 receptor activation, disrupts sodium and fluid imbalance, resulting in hypertension [33].

VEGF is a key regulator of angiogenesis and vascular permeability [34]. Insulin plays a role in increasing *VEGFA* mRNA levels, and local VEGF-A production in the kidney is essential for maintaining the specialized renal vascular network [35]. Insulin resistance impairs podocyte VEGF-A production, thereby disrupting the glomerular filtration barrier, vascular permeability, and angiogenesis [34,35].

The kidneys play a pivotal role in glucose homeostasis by regulating its production, utilization, and excretion [36]. Glucose is freely filtered at the glomerulus and reabsorbed in the proximal tubule under insulin regulation [37]. Sodium-glucose cotransporters (SGLTs), particularly SGLT2, mediate glucose reabsorption, relying on the sodium gradient established by the Na-K ATPase pump [38]. Glycosuria occurs when blood glucose levels exceed the renal threshold, surpassing the tubules’ reabsorptive capacity [39]. In diabetic patients with hyperinsulinemia, early glomerular hyperfiltration triggers early tubular growth and hyperreabsorption via increased SGLT activity, altering tubuloglomerular feedback [40]. Subsequently, the intrarenal activation of the renin–angiotensin–aldosterone system (RAAS) and tubular glycogen accumulation further exacerbate kidney damage [40]. These pathological mechanisms form the basis for the therapeutic efficacy of SGLT2 inhibitors, which may protect against diabetes-induced renal microcirculatory damage.

### 2.3. Abdominal Obesity

Obesity is closely linked to high glucose levels, lipid abnormalities, and hypertension, but this section focuses on abdominal fat accumulation. In abdominal obesity, visceral fat produces pro-inflammatory cytokines, known as adipocytokines, including leptin, resistin, visfatin, interleukin 6 (IL-6), and tumor necrosis factor α (TNF-α), which influence systemic inflammation and insulin sensitivity [41]. TNF-α stimulates nicotinamide adenine dinucleotide phosphate oxidase (NOX), which reduces endothelial NO expression, resulting in renal vascular endothelial dysfunction [42]. Elevated visfatin levels correlate with soluble vascular adhesion molecule 1, indicating vascular damage and endothelial dysfunction [43]. Additionally, NF-κB, a signaling pathway upregulated in obesity and CKD, promotes inflammation and regulates apoptosis and vascular remodeling. However, disrupting NF-κB signaling can reduce inflammation, fibrosis, and microvascular rarefaction, improving renal function in swine CKD with high cholesterol [44]. In uremic conditions, adipocytokine levels rise due to impaired renal excretion, accelerating inflammation [45].

Recent studies show exosomes from visceral fat are associated with endothelial inflammation, activating the NF-κB pathway by targeting the coding sequence region of *peroxisome proliferator-activated receptor alpha (PPARA)* mRNA to endothelial cells. Elevated exosomal microRNA-27b-3p in visceral fat increases the risk of vascular inflammation and atherosclerosis, though these effects have not been directly noted in the kidney [46]. Chronic low-grade inflammation impairs endothelial function, leading to microvascular dysfunction. This inflammation also triggers macrophages and leukocytes to infiltrate adipose tissue, where they release pro-fibrotic cytokines like TGF-Β [47,48,49]. TGF-Β promotes excessive extracellular matrix accumulation, leading to the remodeling of the cortical and medullary renal vascular tree, which contributes to obesity-related kidney disease [50,51].

Visceral fat also generates excessive reactive oxygen species (ROS), increasing oxidative stress, reducing NO production, and damaging endothelial cells, resulting in in hypertension, vascular stiffness, and damage to target organs such as the heart, kidney, and brain [52]. Decreased adiponectin levels in visceral obesity reduce anti-inflammatory responses, impairing kidney microcirculation. Adiponectin is primarily localized to the arteriolar endothelium and platelet-derived growth factor receptor Β-positive pericytes of peritubular capillaries, and it promotes exosome secretion from pericytes to maintain the capillary network [53]. However, in CKD, adiponectin is reported to play a more complex role. Elevated adiponectin levels in CKD are associated with increased mortality, suggesting the need for cautious interpretation and further investigation in obese CKD patients [54].

Obesity impacts renal capillaries by increasing glomerular capillary density while decreasing peritubular capillaries. Adipose tissue preserves a dense vascular network to deliver oxygen and nutrients, where IL-6 levels increase during adipocyte differentiation, corresponding with adipose cell size. Elevated IL-6 in obese individuals promotes VEGF expression, contributing to kidney vascular remodeling and increased GFR in early MetS [55]. In obese pigs with MetS, elevated glomerular hydrostatic pressure promotes hypertrophy, and early renal capillarization is linked to lipid-induced inflammation [56]. VEGF stimulates endothelial cell proliferation and activates sterol regulatory element-binding proteins (SREBPs), which regulate lipid homeostasis. In high-fat diet mice, elevated SREBP expression leads to the renal accumulation of TG and cholesterol, increasing plasminogen activator inhibitor 1 (PAI-1), VEGF, type IV collagen, and fibronectin [57]. Ang II and shear stress from glomerular capillaries activates SREBPs, contributing to vascular development and kidney fibrosis [58].

In contrast, peritubular capillary density decreases due to endothelial cell dysfunction, particularly in obese pigs with renal artery stenosis [56]. In a swine model of MetS, treatment with the mitochondrial cardiolipin-targeting peptide elamipretide improved apoptosis and oxidative stress in peritubular capillary endothelial cells, increasing renal microvascular density and reducing vascular remodeling, suggesting that MetS-induced mitochondrial alterations contribute to renal microvascular loss [59]. Despite these improvements, tubular hypertrophy persists, likely due to increased tubular ultrafiltration resulting from glomerular hypertrophy [13,60].

### 2.4. Dyslipidemia

In MetS, dyslipidemia arises from increased glucose and fatty acids levels due to insulin resistance, leading to lipid accumulation in kidney cells [61]. In diabetic animals, lipid accumulation is apparently observed in both the tubular and glomerular regions [62]. Additionally, excessive fat leads to lipid overflow and ectopic fat deposition in the microvasculature [63].

Lipid accumulation in the kidneys activates various inflammatory signaling pathways, exacerbating renal dysfunction in a process known as lipotoxicity [62]. Toxic lipid metabolites in dyslipidemia alter the cellular redox environment, creating a more oxidized state, which results in the activation of the renin–angiotensin system in the kidneys [64,65,66]. Increased oxidative stress may, in turn, lead to endoplasmic reticulum and mitochondrial dysfunction, as well as inflammatory responses through the NF-κB/Kelch-like ECH-associated protein 1 and nuclear factor erythroid 2-related factor 2 pathway, along with an amplification of apoptosis in the cells of the glomeruli and tubules [67]. The overloading of fatty acids triggers incomplete beta-oxidation, while oxidized (ox)-lipids promote endothelial leukocyte adhesion and migration, increasing vascular permeability and leading to peritubular capillaries damage in rats with MetS [68]. The uptake of low-density lipoprotein (LDL) and ox-LDL by mesangial cells leads to cell proliferation and the expansion of the glomerular matrix [69]. In proximal tubular epithelial cells, this uptake results in an increased expression of extracellular matrix proteins with tubulointerstitial lesions [69]. Furthermore, ox-LDL within the vascular intima contributes to atherosclerosis by triggering endothelial cell dysfunction [61,70].

A recent study in patients with MetS revealed a significant increase in moderate and severe arteriolar hyalinosis and arteriosclerosis affecting the kidney’s arterioles, arteries, and peritubular vessels, compared to those without MetS [71]. This suggests that intrarenal ischemia may play a role in MetS-related kidney injury. Moreover, our group has shown that obesity intensifies ischemia-reperfusion injury in murine kidneys [10]. The kidney’s vulnerability to hypoxia, due to multiple arteriovenous shunts in the intrarenal vasculature, compounds the effects of ischemia in MetS-related kidney injury [72]. Consequently, the ongoing loss of functioning nephrons, exacerbated by renal ischemia and cellular hypoxia, combined with subsequent hypertension and hyperfiltration in the remaining nephrons, may contribute to the development of CKD in obesity and MetS.

### 2.5. Increased Blood Pressure

In MetS and obesity, several factors are linked to increased blood pressure, including physical compression of the kidneys by perirenal fat, the activation of the RAAS, mineralocorticoid receptor (MR) activation independent of aldosterone, and enhanced renal sympathetic nerve activity [73]. Hemodynamic changes related to renal sodium retention, along with the activation of the coagulation system, inflammation, and increased oxidative stress, are key drivers in the development of kidney microvascular dysfunction.

Adipocytes secrete angiotensinogen, which is converted to angiotensin I and subsequently to angII, significantly raising blood pressure [73]. AngII regulates vascular tone and resistance, increasing glomerular hydrostatic pressure by causing the vasoconstriction of the efferent and, to a lesser extent, the afferent arterioles [74]. This triggers tubuloglomerular feedback activation. Combined with the elevation of sympathetic renal nerve activity and RAAS-mediated myogenic regulatory mechanisms, these processes lead to decreased renal perfusion and oxygenation [75]. RAAS is also associated with the activation of the coagulation system [76], which may lead to local defects in microcirculation and localized ischemic changes, resulting in endothelial cell damage and increased vascular permeability [75,77].

Obesity leads to MR activation via aldosterone-independent mechanisms such as increased renal tubular expression of ras-related C3 botulinum toxin substrate 1 and increased ROS [78]. MR activation in vascular smooth muscle and vascular endothelium contributes to glomerular injury via increased blood pressure and then glomerular hyperfiltration [79].

Elevated sympathetic nerve activity in obesity also stimulates renal tubular sodium reabsorption directly and indirectly by promoting renin release and further activating RAAS. This activation increases renal vascular resistance and decreases renal blood flow [80]. The chronic elevation of leptin from visceral adipose tissue also increases sympathetic nerve activity [73]. Leptin affects NO synthesis in the kidneys and promotes arterial relaxation by enhancing NO bioavailability. However, in obesity-related endothelial dysfunction, the inhibition of NO release impairs leptin’s role in regulating blood pressure, contributing to hypertension [73,81].

**Figure 1:**
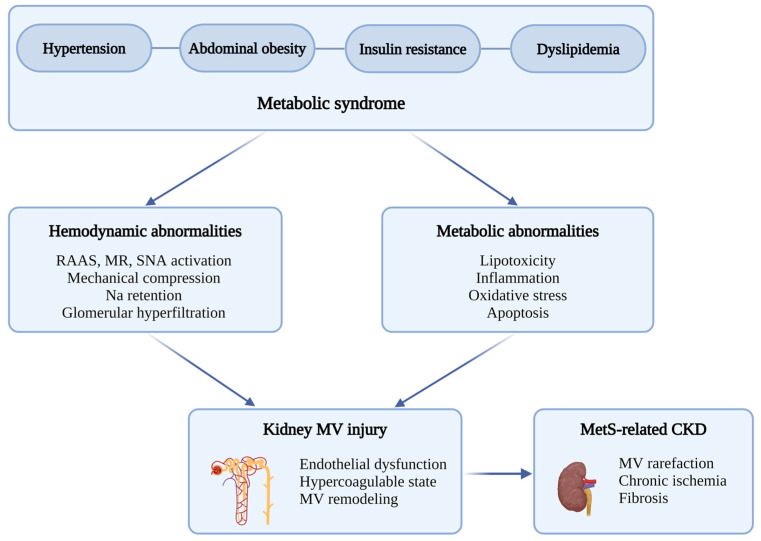
Mechanisms through which metabolic syndrome (MetS) leads to kidney microvascular injury. MetS, characterized by hypertension, abdominal obesity, insulin resistance, and dyslipidemia, results in hemodynamic and metabolic abnormalities. These abnormalities lead to the activation of RAAS, MR, and SNA, mechanical compression, sodium retention, and glomerular hyperfiltration, as well as lipotoxicity, inflammation, oxidative stress, and apoptosis. Together, these factors contribute to kidney microvascular injury, including endothelial dysfunction, a hypercoagulable state, and microvascular remodeling, ultimately leading to MetS-related CKD. Abbreviations: CKD, chronic kidney disease; MetS, metabolic syndrome; MR, mineralocorticoid receptor; MV, microvascular; RAAS, renin–angiotensin–aldosterone system; SNA, sympathetic nerve activity. Created in https://BioRender.com.

The mechanisms by which individual components of MetS affect kidney microcirculation are interconnected and act synergistically, accelerating kidney microvascular injury (Figure 1). Insulin resistance, hypertension, and dyslipidemia contribute to endothelial dysfunction by impairing NO production and increasing oxidative stress, leading to sustained vasoconstriction. Simultaneously, hypertension and hyperglycemia promote vascular remodeling, which results in a thickening of vessel walls and increased stiffness, thereby reducing blood flow to the renal microcirculation. This combination of endothelial dysfunction and vascular remodeling leads to renal microvascular rarefaction, decreasing oxygen and nutrient delivery to kidney tissues. Additionally, the accumulation of lipids in ectopic tissues exerts lipotoxic effects that further damage the kidneys. Chronic inflammation, driven by adipokines and pro-inflammatory cytokines from visceral fat, exacerbates these detrimental processes. Collectively, these factors—endothelial dysfunction, vascular remodeling, and microvascular rarefaction—contribute to the progression of kidney damage and CKD in MetS.

## 3. Kidney-Related Adiposity and Microcirculation

**Figure 2:**
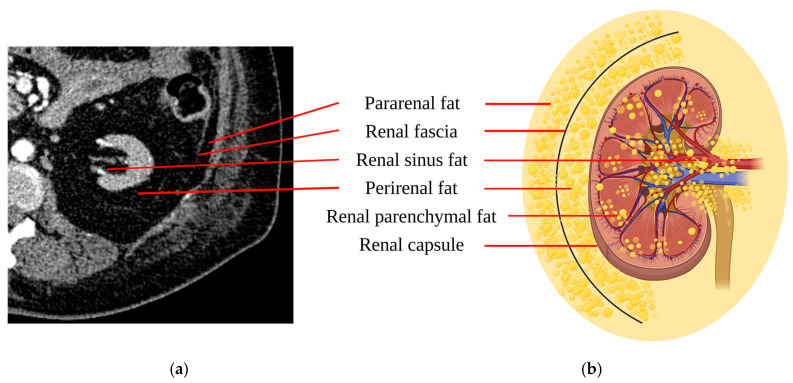
Kidney-related adiposity. (**a**) Computed tomography image of an obese subject (BMI 30.5 kg/m^2^) with advanced chronic kidney disease, highlighting different fat deposits around the kidney. (**b**) Schematic illustration of ectopic kidney fat showing anatomical localization of renal fascia, pararenal fat, renal sinus fat, renal capsule, perirenal fat, and renal parenchymal fat [105]. Created in https://BioRender.com.

Ectopic kidney fat is associated with the development of diabetes, hypertension, and CKD, leading to its proposal as a distinct clinical entity termed “fatty kidney disease”, akin to fatty liver disease [82]. While kidney-related fat is often conflated, it is anatomically and physiologically distinct (Figure 2). Perirenal fat surrounds the kidneys between the renal fibrous membrane and fascia, while pararenal fat, located next to it, is separated by the renal fascia. RSF, on the other hand, is perivascular fat within the renal sinus and is considered part of perirenal fat. It is marked by a straight line across the renal sinus opening, connecting the indentations of adjacent lobes. Perirenal fat has a distinct blood supply, lymph drainage, and innervation compared to typical connective tissues [83]. Histologically, pararenal fat is white adipose tissue, while perirenal fat primarily contains dormant brown adipose tissue, which is more active in energy metabolism and adipokine secretion [84]. Renal parenchymal fat is located within the renal cortex and medulla.

Perirenal fat was thicker in obese patients with microalbuminuria than in those without, suggesting that perirenal fat thickness may be an independent predictor of early kidney damage in obese individuals [85]. In pigs with early MetS, perirenal fat has been shown to directly cause renal arterial endothelial dysfunction, partly mediated by TNF-α [42]. This dysfunction is likely due to increased oxidative stress and the activation of inflammatory molecular pathways resulting from elevated free fatty acids and low adiponectin levels [86]. Recent studies highlight perirenal fat as a significant factor in cardiovascular risk, distinct from subcutaneous fat [87,88]. Excess perirenal fat is associated with cardiometabolic risk factors, including WC, TG, HDL cholesterol, and insulin resistance [89]. Furthermore, Campobasso et al. reported a positive correlation between perirenal fat thickness and mean daily diastolic blood pressure [90]. Its metabolic activity, including the secretion of pro-inflammatory cytokines and adipokines, contributes to inflammation and endothelial dysfunction [91,92]. Additionally, increased perirenal fat thickness has been linked to elevated cardiovascular risk predictions in individuals with type 2 diabetes, as assessed by the Framingham Risk Score [91]. These findings underscore the significance of perirenal fat as an independent cardiovascular risk factor, deserving further research and clinical focus.

Similarly, pararenal fat has been linked to visceral obesity and renal dysfunction, even in patients without clinically significant cardiovascular diseases (CVD) [93]. Significant correlations have been observed between pararenal fat thickness and anthropometric obesity criteria, such as BMI and WC, as well as free fatty acids [94,95]. The mechanisms of lipotoxicity-related kidney damage in obesity involve elevated circulating free fatty acids that impair endothelial function by the uncoupling of the VEGF–NO axis [95]. This contributes to endothelial cell proliferation, increased permeability, and microalbuminuria.

RSF accumulation is linked to renal dysfunction and intrarenal hemodynamic abnormalities, independent of visceral adiposity [96]. RSF accumulation compresses arterial and venous blood flow, as well as nerve bundles due to the absence of a renal capsule at the hilum. This compression reduces arterial inflow and increases intrarenal venous pressure, potentially lowering estimated GFR (eGFR) [97]. Additionally, the lack of a hilar capsule facilitates the extension of extrarenal fat into the renal medullary space [97]. Recent studies have shown that RSF is strongly correlated with the renal resistive index, which is associated with microvascular remodeling [96,98]. Accumulated RSF may compress the thin loop of Henle and vasa recta, reducing tubular and medullary blood flow, leading to increased sodium reabsorption in the thick loop of Henle. This lowers sodium levels in the distal tubule, prompting the macula densa to trigger afferent arteriole dilation, leading to glomerular hyperfiltration and contributing to the progression of CKD [99]. This compression also activates the RAAS and increases sympathetic nervous system activity [100]. Furthermore, RSF also contributes to lipotoxicity, inflammation, oxidative stress, and renal fibrosis, exacerbating disruptions in kidney microcirculation [67].

Ectopic renal parenchymal fat also leads to lipotoxicity, resulting in albuminuria and CKD, along with systemic effects [101]. A previous study using magnetic resonance imaging to assess the renal parenchymal fat fraction has shown that patients in the highest tertile of the renal parenchymal fat fraction have the greatest risk of CKD compared to those in the lowest tertile [102]. However, current studies have shown that interventions such as dietary weight loss or exercise may not effectively reduce renal parenchymal fat [103,104].

## 4. Therapeutic Approaches

### 4.1. Anthropometric and Laboratory-Based Cardiovascular Risk Indices

In managing MetS, optimizing cardiovascular risk indices derived from anthropometric or laboratory parameters is an important therapeutic objective, particularly in settings where advanced imaging is unavailable. Indices like BMI, WC, waist-to-hip ratio, visceral adiposity index, the TG–glucose (TyG) index, and newer metrics such as the body roundness index (BRI), lipid accumulation product (LAP), and a body shape index (ABSI) provide valuable markers of cardiometabolic risk [106,107,108,109,110,111]. While BMI and WC are widely used, they have limitations in evaluating visceral fat and adipose distribution, leading to the development of more comprehensive models such as Samouda et al.’s visceral adipose tissue index, which incorporates WC, thigh circumferences, age, and BMI [112]. This index has been shown to outperform BMI and WC alone in predicting cardiometabolic abnormalities and mortality risk [112]. ABSI and BRI enhance the assessment of body shape and roundness, with studies showing their superior predictive value for CVD and mortality across diverse populations [106,111]. The waist-to-height ratio excels in predicting dyslipidemia, hyperglycemia, and CVD [106], while the weight-adjusted waist index is effective for assessing sarcopenic obesity and cardiometabolic risks in elderly populations [107]. Indices such as LAP, which combines WC and TG, and TyG, which uses fasting plasma TG and glucose, effectively predict MetS, insulin resistance, and cardiovascular risks [113]. The TyG index, particularly in large cohorts such as the PURE study, has demonstrated strong associations with future cardiovascular mortality, myocardial infarction, stroke, and type 2 diabetes, highlighting its utility in understanding insulin resistance and its role in the pathogenesis of MetS and CVD [109,110]. However, the relevance and accuracy of these indices may vary across different population groups, highlighting the need for cautious interpretation and the consideration of population-specific characteristics.

### 4.2. Current Pharmacological Interventions

Although no medications are specifically approved for treating MetS itself, drugs targeting each individual component, such as antihypertensives, antidiabetics, and lipid-lowering agents, should be addressed early and properly.

Angiotensin-converting enzyme (ACE) inhibitors and AngII receptor blockers (ARBs) reduce glomerular hypertension and microvascular injury by promoting renal vasodilation and enhancing renal blood flow, thereby mitigating ischemia and hypoxia in the kidney [114,115]. The expression of angiotensinogen, ACE, and AT1 receptors is higher in visceral adipose tissue compared to subcutaneous adipose tissue [116]. Additionally, ACE inhibitors and ARBs enhance insulin sensitivity and promote natriuresis, resulting in decreased glomerular hyperfiltration and hyperperfusion in obese hypertensive rodents [116,117]. MR antagonists effectively lower blood pressure in treatment-resistant obese patients. Their use alongside ACE inhibitors or ARBs significantly reduces blood pressure, suggesting MR activation occurs independently of AngII-mediated aldosterone secretion [73]. Short-term MR antagonist treatment can rapidly correct glomerular hyperfiltration, reducing albuminuria, although it may temporarily decrease eGFR before stabilizing [118]. However, the long-term impact of MR antagonists on kidney microcirculation in MetS requires further investigation.

Metformin has substantial evidence supporting its direct beneficial effects on endothelial function. Metformin restores endothelial function by inhibiting endoplasmic reticulum stress and oxidative stress and increasing NO bioavailability through the activation of the AMP-activated protein kinase (AMPK)/PPARδ pathway, along with potential AMPK-independent mechanisms [119,120]. In subjects with type 2 diabetes and CKD, linagliptin, a dipeptidyl peptidase-4 inhibitor, has demonstrated improvements in endothelial dysfunction when used in combination with metformin and/or insulin [121]. This improvement is associated with an enhanced migratory function of cluster of differentiation 34 (CD34) positive endothelial progenitor cells, as indicated by increased CD34/C-X-C chemokine receptor type 4 positivity [121]. Empagliflozin, an SGLT2 inhibitor, has shown renoprotective properties by reducing endothelial apoptosis, enhancing NO production, and preventing microvascular damage [122]. Similarly, Dapagliflozin improves endothelium-dependent vasorelaxation in diabetic mice and attenuates endothelial dysfunction via the sirtuin1–endothelial NO synthase axis [123]. SGLT2 inhibitors exhibit anti-apoptotic, anti-inflammatory, and oxidative stress-reducing properties, which enhance cerebral microvascular circulation and may provide neuroprotective benefits for patients at risk of cognitive impairment due to type 2 diabetes [124,125]. Additionally, their antiplatelet and antithrombotic effects, linked to NOX2 pathway downregulation, further support vascular and cognitive health [29]. Glucagon-like peptide-1 (GLP-1) protects endothelial NO synthase function against the detrimental effects of hyperglycemia, potentially reducing TNF-α-induced expression of PAI-1 [126,127]. GLP-1 receptor agonists, such as liraglutide, induce distinct transcriptional changes in kidney endothelial cells, which are associated with pathways involved in nutrient utilization, reduction–oxidation (redox) sensing, and the resolution of inflammation [128].

Lipid-lowering agents reduce lipid accumulation in the tubulointerstitial and glomerular areas of the kidney, thereby decreasing inflammation and oxidative stress [129]. Omega-3 fatty acids suppress NF-κB-driven inflammation and modulate inflammasome signaling in CKD patients [130,131]. Statins prevent podocyte apoptosis and nephrin loss through the activation of the p85 PI3K/Akt pathway [132]. Fenofibrate reduces lipid toxicity, tubular cell apoptosis, and albuminuria by activating the AMPK-PPARγ coactivator 1-α signaling pathway, benefiting patients with diabetic kidney disease without adverse effects on renal outcomes [133,134,135]. Ezetimibe activates lipid catabolism and inhibits RAAS and TGF-Β1 pathways, decreasing lipid accumulation in podocytes, endothelial cells, and tubular cells and reducing albuminuria, inflammation, and fibrosis [61,136,137]. Combining simvastatin with ezetimibe effectively lowers LDL cholesterol but shows limited benefits for CKD progression in the general population [138]. Evidence suggests ezetimibe inhibits nucleotide-binding oligomerization domain-like receptor family pyrin domain containing 3 inflammasome activation in macrophages, offering renoprotective effects [137,139]. Recent studies indicate that combining ezetimibe with statins significantly reduces the incidence of adverse renal events compared to statins alone [140]. Proprotein convertase subtilisin/kexin type 9 (PCSK9) inhibitors lower LDL cholesterol by promoting LDL receptor degradation and reducing lipid influx through decreased CD36 expression, a transmembrane protein that functions in lipid uptake, inflammation, and fibrosis in renal cells [67,141]. In a high-fat diet murine model, PCSK9 inhibitors reduce renal lipotoxicity and ameliorate renal fibrosis by regulating fatty acid beta-oxidation [142].

### 4.3. Bioactive Agents

Pyridoxamine, a vitamin B6 analog, has demonstrated a vasoprotective effect on renal endothelial dysfunction in early MetS-related kidney injury [143]. A recent study showed that high-fat diet-feeding in a MetS animal model induced paradoxical vasoconstriction in renal arteries, whereas pyridoxamine significantly improved kidney vasorelaxation, likely due to its metabolic and antioxidant properties [143]. Natural bioactive compounds have demonstrated a positive role in the clinical management of MetS and its comorbidities, by improving body weight, blood pressure, glucose metabolism, endothelial function, lipid profile, inflammation, and oxidative stress [144]. Several studies revealed that curcumin protects the kidneys from oxidative stress and inflammation, improving endothelial function [145]. In hypertriglyceridemia rats, curcumin reduced lipid synthesis and uptake in the kidneys by improving beta-oxidation disorders in the kidney [146]. Quercetin, a flavonoid, has shown renoprotective effects through inhibiting ferroptosis-triggered renal injury in diabetic nephropathy [147]. Quercetin also improves renal function and partially enhances renal cortical oxygenation, which is associated with alleviating renal senescence in mice on a high-fat diet [148]. Berberine, a natural alkaloid, has demonstrated efficacy and safety on MetS through multiple pathways and targets [149]. In spontaneously hypertensive rats, berberine improved endothelial function by enhancing endothelium-dependent vasodilation and preserving arterial elasticity, as evidenced by reduced aortic pulse wave velocity and increased elastin fiber content in the arterial media [150]. Additionally, berberine alleviates kidney damage by improving the high glucose-induced reduction of fatty acid beta-oxidation in the diabetic kidney [151]. These bioactive agents require further large-scale trials to clarify their mechanisms in metabolic regulation and to establish guidelines for the minimum effective dose and treatment duration for optimal benefits.

## 5. Conclusions and Perspectives

This review highlights the complex role of each MetS component in the development of kidney microvascular injury. The interactions among insulin resistance, hypertension, dyslipidemia, and visceral adiposity initiate a series of hemodynamic and metabolic disruptions that ultimately impair kidney microcirculation. The resulting outcomes—endothelial dysfunction, vascular remodeling, and microvascular rarefaction—substantially drive CKD progression in patients with MetS.

Controlling MetS effectively, especially in view of hypertension, dyslipidemia, and insulin resistance at an early stage, is important to protecting the kidneys. Addressing these factors through targeted pharmacological therapies—such as antihypertensives, lipid-lowering agents, and antidiabetic medications—has shown beneficial outcomes on the microvasculature of the kidney, mainly inflammation and oxidative stress. However, these treatments do not stop renal microvascular injury directly. Therapies that target the improvement of microvascular function or target the improvement of microvasculature by neovascularization or the inhibition of vascular remodeling are currently being researched. In addition to focusing on separate components of MetS, it is also important to target visceral fat since this helps in metabolic risk reduction and optimal kidney microcirculation.

We acknowldege limitations in this study. This review adopts a traditional narrative format, synthesizing key themes and insights, rather than employing a scoping or systematic review. While this format facilitates a broad exploration of relevant topics, it may lack the transparency and systematic processes of other review types, potentially introducing selection bias. Future studies using systematic methodologies could build on these findings to provide a more comprehensive and rigorous evidence synthesis.

The complexity of MetS and its effects on the kidneys may need multiple therapeutic approaches to address different mechanisms effectively, optimizing personalized medicine to enhance renal outcomes and slow CKD progression.

## References

[1] Lemieux I., Despres J.P. (2020). Metabolic Syndrome: Past, Present and Future. Nutrients.

[2] Alberti K.G., Eckel R.H., Grundy S.M., Zimmet P.Z., Cleeman J.I., Donato K.A., Fruchart J.C., James W.P., Loria C.M., Smith S.C. (2009). Harmonizing the metabolic syndrome: A joint interim statement of the International Diabetes Federation Task Force on Epidemiology and Prevention; National Heart, Lung, and Blood Institute; American Heart Association; World Heart Federation; International Atherosclerosis Society; and International Association for the Study of Obesity. Circulation.

[3] Christian Flemming G.M., Bussler S., Korner A., Kiess W. (2020). Definition and early diagnosis of metabolic syndrome in children. J. Pediatr. Endocrinol. Metab..

[4] Zimmet P., Alberti K.G., Kaufman F., Tajima N., Silink M., Arslanian S., Wong G., Bennett P., Shaw J., Caprio S. (2007). The metabolic syndrome in children and adolescents—An IDF consensus report. Pediatr. Diabetes.

[5] Powell-Wiley T.M., Poirier P., Burke L.E., Despres J.P., Gordon-Larsen P., Lavie C.J., Lear S.A., Ndumele C.E., Neeland I.J., Sanders P. (2021). Obesity and Cardiovascular Disease: A Scientific Statement From the American Heart Association. Circulation.

[6] Ritchie S.A., Connell J.M. (2007). The link between abdominal obesity, metabolic syndrome and cardiovascular disease. Nutr. Metab. Cardiovasc. Dis..

[7] Antonio-Villa N.E., Bello-Chavolla O.Y., Vargas-Vazquez A., Mehta R., Fermin-Martinez C.A., Martagon-Rosado A.J., Barquera-Guevara D.A., Aguilar-Salinas C.A., Metabolic Syndrome Study G. (2021). Increased visceral fat accumulation modifies the effect of insulin resistance on arterial stiffness and hypertension risk. Nutr. Metab. Cardiovasc. Dis..

[8] Hwang Y.C., Fujimoto W.Y., Hayashi T., Kahn S.E., Leonetti D.L., Boyko E.J. (2016). Increased Visceral Adipose Tissue Is an Independent Predictor for Future Development of Atherogenic Dyslipidemia. J. Clin. Endocrinol. Metab..

[9] Grigoraș A., Balan R.A., Căruntu I.D., Giușcă S.E., Lozneanu L., Avadanei R.E., Rusu A., Riscanu L.A., Amalinei C. (2021). Perirenal Adipose Tissue-Current Knowledge and Future Opportunities. J. Clin. Med..

[10] Kim S.R., Kim Y.S., Hyeon J.M., Kim S.J., Ye B.M., Kim M.J., Choi B.H., Yi D., Kim I.Y., Lee S.B. (2024). Obesity exacerbates ischemia-reperfusion injury and senescence in murine kidneys and perirenal adipose tissues. Kidney Res. Clin. Pract..

[11] Foster M.C., Hwang S.J., Porter S.A., Massaro J.M., Hoffmann U., Fox C.S. (2011). Fatty kidney, hypertension, and chronic kidney disease: The Framingham Heart Study. Hypertension.

[12] Lin L., Tan W., Pan X., Tian E., Wu Z., Yang J. (2022). Metabolic Syndrome-Related Kidney Injury: A Review and Update. Front. Endocrinol..

[13] Ye M., Yang M., Dai W., Li H., Zhou X., Chen Y., He L. (2023). Targeting Renal Proximal Tubule Cells in Obesity-Related Glomerulopathy. Pharmaceuticals.

[14] Chade A.R. (2013). Renal vascular structure and rarefaction. Compr. Physiol..

[15] Wilcox C.S., Welch W.J., Murad F., Gross S.S., Taylor G., Levi R., Schmidt H.H. (1992). Nitric oxide synthase in macula densa regulates glomerular capillary pressure. Proc. Natl. Acad. Sci. USA.

[16] Carlstrom M., Wilcox C.S., Arendshorst W.J. (2015). Renal autoregulation in health and disease. Physiol. Rev..

[17] Burke M., Pabbidi M.R., Farley J., Roman R.J. (2014). Molecular mechanisms of renal blood flow autoregulation. Curr. Vasc. Pharmacol..

[18] Fan L., Gao W., Nguyen B.V., Jefferson J.R., Liu Y., Fan F., Roman R.J. (2020). Impaired renal hemodynamics and glomerular hyperfiltration contribute to hypertension-induced renal injury. Am. J. Physiol. Renal Physiol..

[19] Zafrani L., Ince C. (2015). Microcirculation in Acute and Chronic Kidney Diseases. Am. J. Kidney Dis..

[20] Ribiere C., Jaubert A.M., Sabourault D., Lacasa D., Giudicelli Y. (2002). Insulin stimulates nitric oxide production in rat adipocytes. Biochem. Biophys. Res. Commun..

[21] Tsukahara H., Kikuchi K., Tsumura K., Kimura K., Hata I., Hiraoka M., Sudo M. (1997). Experimentally induced acute hyperinsulinemia stimulates endogenous nitric oxide production in humans: Detection using urinary NO_2_-/NO_3_-excretion. Metabolism.

[22] Villa E., Garcia-Robles R., Romero J.C. (1998). Effects of hyperinsulinemia on the regulation of regional blood flow and blood pressure in anesthetized dogs: Hemodynamic role of nitric oxide. Am. J. Hypertens..

[23] Komers R., Pelikanova T., Kazdova L. (1999). Effect of hyperinsulinaemia on renal function and nitrate/nitrite excretion in healthy subjects. Clin. Exp. Pharmacol. Physiol..

[24] Hu R.M., Levin E.R., Pedram A., Frank H.J. (1993). Insulin stimulates production and secretion of endothelin from bovine endothelial cells. Diabetes.

[25] Rivera-Gonzalez O.J., Kasztan M., Johnston J.G., Hyndman K.A., Speed J.S. (2020). Loss of endothelin type B receptor function improves insulin sensitivity in rats. Can. J. Physiol. Pharmacol..

[26] Rivera-Gonzalez O., Mills M.F., Konadu B.D., Wilson N.A., Murphy H.A., Newberry M.K., Hyndman K.A., Garrett M.R., Webb D.J., Speed J.S. (2024). Adipocyte endothelin B receptor activation inhibits adiponectin production and causes insulin resistance in obese mice. Acta Physiol..

[27] Rivera-Gonzalez O., Wilson N.A., Coats L.E., Taylor E.B., Speed J.S. (2021). Endothelin receptor antagonism improves glucose handling, dyslipidemia, and adipose tissue inflammation in obese mice. Clin. Sci..

[28] Nakamura T., Ebihara I., Fukui M., Tomino Y., Koide H. (1995). Effect of a specific endothelin receptor A antagonist on mRNA levels for extracellular matrix components and growth factors in diabetic glomeruli. Diabetes.

[29] Wang X.H., Ao Q.G., Cheng Q.L. (2021). Caloric restriction inhibits renal artery ageing by reducing endothelin-1 expression. Ann. Transl. Med..

[30] Ahmad Banday A., Lokhandwala M.F. (2006). Defective renal dopamine D1 receptor function contributes to hyperinsulinemia-mediated hypertension. Clin. Exp. Hypertens..

[31] Wehbi G.J., Zimpelmann J., Carey R.M., Levine D.Z., Burns K.D. (2001). Early streptozotocin-diabetes mellitus downregulates rat kidney AT2 receptors. Am. J. Physiol. Renal Physiol..

[32] Cheng H.F., Burns K.D., Harris R.C. (1994). Reduced proximal tubule angiotensin II receptor expression in streptozotocin-induced diabetes mellitus. Kidney Int..

[33] Hussain T. (2003). Renal angiotensin II receptors, hyperinsulinemia, and obesity. Clin. Exp. Hypertens..

[34] Hale L.J., Hurcombe J., Lay A., Santamaria B., Valverde A.M., Saleem M.A., Mathieson P.W., Welsh G.I., Coward R.J. (2013). Insulin directly stimulates VEGF-A production in the glomerular podocyte. Am. J. Physiol. Renal Physiol..

[35] Eremina V., Baelde H.J., Quaggin S.E. (2007). Role of the VEGF–a signaling pathway in the glomerulus: Evidence for crosstalk between components of the glomerular filtration barrier. Nephron Physiol..

[36] Alsahli M., Gerich J.E. (2017). Renal glucose metabolism in normal physiological conditions and in diabetes. Diabetes Res. Clin. Pract..

[37] Cersosimo E., Garlick P., Ferretti J. (1999). Insulin regulation of renal glucose metabolism in humans. Am. J. Physiol..

[38] Wright E.M., Loo D.D., Hirayama B.A. (2011). Biology of human sodium glucose transporters. Physiol. Rev..

[39] Johansen K., Svendsen P.A., Lorup B. (1984). Variations in renal threshold for glucose in Type 1 (insulin-dependent) diabetes mellitus. Diabetologia.

[40] Vallon V., Komers R. (2011). Pathophysiology of the diabetic kidney. Compr. Physiol..

[41] Kirichenko T.V., Markina Y.V., Bogatyreva A.I., Tolstik T.V., Varaeva Y.R., Starodubova A.V. (2022). The Role of Adipokines in Inflammatory Mechanisms of Obesity. Int. J. Mol. Sci..

[42] Ma S., Zhu X.Y., Eirin A., Woollard J.R., Jordan K.L., Tang H., Lerman A., Lerman L.O. (2016). Perirenal Fat Promotes Renal Arterial Endothelial Dysfunction in Obese Swine through Tumor Necrosis Factor-alpha. J. Urol..

[43] Qiu X., Lan X., Li L., Chen H., Zhang N., Zheng X., Xie X. (2024). The role of perirenal adipose tissue deposition in chronic kidney disease progression: Mechanisms and therapeutic implications. Life Sci..

[44] Chade A.R., Williams M.L., Engel J.E., Williams E., Bidwell G.L. (2020). Molecular targeting of renal inflammation using drug delivery technology to inhibit NF-kappaB improves renal recovery in chronic kidney disease. Am. J. Physiol. Renal Physiol..

[45] Roubicek T., Bartlova M., Krajickova J., Haluzikova D., Mraz M., Lacinova Z., Kudla M., Teplan V., Haluzik M. (2009). Increased production of proinflammatory cytokines in adipose tissue of patients with end-stage renal disease. Nutrition.

[46] Tang Y., Yang L.J., Liu H., Song Y.J., Yang Q.Q., Liu Y., Qian S.W., Tang Q.Q. (2023). Exosomal miR-27b-3p secreted by visceral adipocytes contributes to endothelial inflammation and atherogenesis. Cell Rep..

[47] Decleves A.E., Sharma K. (2015). Obesity and kidney disease: Differential effects of obesity on adipose tissue and kidney inflammation and fibrosis. Curr. Opin. Nephrol. Hypertens..

[48] Kochumon S., Al Madhoun A., Al-Rashed F., Thomas R., Sindhu S., Al-Ozairi E., Al-Mulla F., Ahmad R. (2020). Elevated adipose tissue associated IL-2 expression in obesity correlates with metabolic inflammation and insulin resistance. Sci. Rep..

[49] Makki K., Froguel P., Wolowczuk I. (2013). Adipose tissue in obesity-related inflammation and insulin resistance: Cells, cytokines, and chemokines. ISRN Inflamm..

[50] Decleves A.E., Mathew A.V., Cunard R., Sharma K. (2011). AMPK mediates the initiation of kidney disease induced by a high-fat diet. J. Am. Soc. Nephrol..

[51] Chade A.R., Hall J.E. (2016). Role of the Renal Microcirculation in Progression of Chronic Kidney Injury in Obesity. Am. J. Nephrol..

[52] Koenen M., Hill M.A., Cohen P., Sowers J.R. (2021). Obesity, Adipose Tissue and Vascular Dysfunction. Circ. Res..

[53] Tsugawa-Shimizu Y., Fujishima Y., Kita S., Minami S., Sakaue T.A., Nakamura Y., Okita T., Kawachi Y., Fukada S., Namba-Hamano T. (2021). Increased vascular permeability and severe renal tubular damage after ischemia-reperfusion injury in mice lacking adiponectin or T-cadherin. Am. J. Physiol. Endocrinol. Metab..

[54] Menon V., Li L., Wang X., Greene T., Balakrishnan V., Madero M., Pereira A.A., Beck G.J., Kusek J.W., Collins A.J. (2006). Adiponectin and mortality in patients with chronic kidney disease. J. Am. Soc. Nephrol..

[55] Rega G., Kaun C., Demyanets S., Pfaffenberger S., Rychli K., Hohensinner P.J., Kastl S.P., Speidl W.S., Weiss T.W., Breuss J.M. (2007). Vascular endothelial growth factor is induced by the inflammatory cytokines interleukin-6 and oncostatin m in human adipose tissue in vitro and in murine adipose tissue in vivo. Arterioscler. Thromb. Vasc. Biol..

[56] Li Z., Woollard J.R., Wang S., Korsmo M.J., Ebrahimi B., Grande J.P., Textor S.C., Lerman A., Lerman L.O. (2011). Increased glomerular filtration rate in early metabolic syndrome is associated with renal adiposity and microvascular proliferation. Am. J. Physiol. Renal Physiol..

[57] Jiang T., Wang Z., Proctor G., Moskowitz S., Liebman S.E., Rogers T., Lucia M.S., Li J., Levi M. (2005). Diet-induced obesity in C57BL/6J mice causes increased renal lipid accumulation and glomerulosclerosis via a sterol regulatory element-binding protein-1c-dependent pathway. J. Biol. Chem..

[58] Dorotea D., Koya D., Ha H. (2020). Recent Insights Into SREBP as a Direct Mediator of Kidney Fibrosis via Lipid-Independent Pathways. Front. Pharmacol..

[59] Eirin A., Hedayat A.F., Ferguson C.M., Textor S.C., Lerman A., Lerman L.O. (2018). Mitoprotection preserves the renal vasculature in porcine metabolic syndrome. Exp. Physiol..

[60] Chagnac A., Zingerman B., Rozen-Zvi B., Herman-Edelstein M. (2019). Consequences of Glomerular Hyperfiltration: The Role of Physical Forces in the Pathogenesis of Chronic Kidney Disease in Diabetes and Obesity. Nephron.

[61] Mitrofanova A., Merscher S., Fornoni A. (2023). Kidney lipid dysmetabolism and lipid droplet accumulation in chronic kidney disease. Nat. Rev. Nephrol..

[62] Thongnak L., Pongchaidecha A., Lungkaphin A. (2020). Renal Lipid Metabolism and Lipotoxicity in Diabetes. Am. J. Med. Sci..

[63] Ji T., Fang B., Wu F., Liu Y., Cheng L., Li Y., Wang R., Zhu L. (2023). Diet Change Improves Obesity and Lipid Deposition in High-Fat Diet-Induced Mice. Nutrients.

[64] Singh B.M., Mehta J.L. (2003). Interactions between the renin-angiotensin system and dyslipidemia-Relevance in the therapy of hypertension and coronary heart disease. Arch. Intern. Med..

[65] Ma K.L., Ni J., Wang C.X., Liu J., Zhang Y., Wu Y., Lv L.L., Ruan X.Z., Liu B.C. (2013). Interaction of RAS activation and lipid disorders accelerates the progression of glomerulosclerosis. Int. J. Med. Sci..

[66] Bagby S.P. (2004). Obesity-initiated metabolic syndrome and the kidney: A recipe for chronic kidney disease?. J. Am. Soc. Nephrol..

[67] Chae S.Y., Kim Y., Park C.W. (2023). Oxidative Stress Induced by Lipotoxicity and Renal Hypoxia in Diabetic Kidney Disease and Possible Therapeutic Interventions: Targeting the Lipid Metabolism and Hypoxia. Antioxidants.

[68] Temm C., Dominguez J.H. (2007). Microcirculation: Nexus of comorbidities in diabetes. Am. J. Physiol. Renal Physiol..

[69] Lin P.H., Duann P. (2020). Dyslipidemia in Kidney Disorders: Perspectives on Mitochondria Homeostasis and Therapeutic Opportunities. Front. Physiol..

[70] Malekmohammad K., Bezsonov E.E., Rafieian-Kopaei M. (2021). Role of Lipid Accumulation and Inflammation in Atherosclerosis: Focus on Molecular and Cellular Mechanisms. Front. Cardiovasc. Med..

[71] Rodriguez-Rodriguez R., Hornum M., Rodriguez Rodriguez A.E., Bevc S., Trevisani F., Fernandez G., Hojs R., Fernandez-Fernandez B., Cases Corona C.M., Cruzado J.M. (2024). Renal Disease in Metabolic Syndrome: The Hidden Role of Intrarenal Ischemia. Kidney Int. Rep..

[72] Mimura I., Nangaku M. (2010). The suffocating kidney: Tubulointerstitial hypoxia in end-stage renal disease. Nat. Rev. Nephrol..

[73] Hall J.E., do Carmo J.M., da Silva A.A., Wang Z., Hall M.E. (2015). Obesity-induced hypertension: Interaction of neurohumoral and renal mechanisms. Circ. Res..

[74] Treeck B., Roald A.B., Tenstad O., Aukland K. (2002). Effect of exogenous and endogenous angiotensin II on intrarenal distribution of glomerular filtration rate in rats. J. Physiol..

[75] Sun D., Wang J., Shao W., Wang J., Yao L., Li Z., Ohno S. (2020). Pathogenesis and Damage Targets of Hypertensive Kidney Injury. J. Transl. Int. Med..

[76] Senchenkova E.Y., Russell J., Esmon C.T., Granger D.N. (2014). Roles of Coagulation and fibrinolysis in angiotensin II-enhanced microvascular thrombosis. Microcirculation.

[77] Xu S., Ilyas I., Little P.J., Li H., Kamato D., Zheng X., Luo S., Li Z., Liu P., Han J. (2021). Endothelial Dysfunction in Atherosclerotic Cardiovascular Diseases and Beyond: From Mechanism to Pharmacotherapies. Pharmacol. Rev..

[78] Shibata S., Nagase M., Yoshida S., Kawachi H., Fujita T. (2007). Podocyte as the target for aldosterone: Roles of oxidative stress and Sgk1. Hypertension.

[79] Fujita T. (2010). Mineralocorticoid receptors, salt-sensitive hypertension, and metabolic syndrome. Hypertension.

[80] Hall J.E., Brands M.W., Henegar J.R. (1999). Mechanisms of hypertension and kidney disease in obesity. Ann. N. Y. Acad. Sci..

[81] Aizawa-Abe M., Ogawa Y., Masuzaki H., Ebihara K., Satoh N., Iwai H., Matsuoka N., Hayashi T., Hosoda K., Inoue G. (2000). Pathophysiological role of leptin in obesity-related hypertension. J. Clin. Investig..

[82] Mende C.W., Einhorn D. (2019). Fatty Kidney Disease: A New Renal and Endocrine Clinical Entity? Describing the Role of the Kidney in Obesity, Metabolic Syndrome, and Type 2 Diabetes. Endocr. Pract..

[83] Czaja K., Kraeling R., Klimczuk M., Franke-Radowiecka A., Sienkiewicz W., Lakomy M. (2002). Distribution of ganglionic sympathetic neurons supplying the subcutaneous, perirenal and mesentery fat tissue depots in the pig. Acta Neurobiol. Exp..

[84] Jespersen N.Z., Feizi A., Andersen E.S., Heywood S., Hattel H.B., Daugaard S., Peijs L., Bagi P., Feldt-Rasmussen B., Schultz H.S. (2019). Heterogeneity in the perirenal region of humans suggests presence of dormant brown adipose tissue that contains brown fat precursor cells. Mol. Metab..

[85] D’Marco L., Salazar J., Cortez M., Salazar M., Wettel M., Lima-Martínez M., Rojas E., Roque W., Bermúdez V. (2019). Perirenal fat thickness is associated with metabolic risk factors in patients with chronic kidney disease. Kidney Res. Clin. Pract..

[86] Hou N., Han F., Wang M., Huang N., Zhao J., Liu X., Sun X. (2014). Perirenal fat associated with microalbuminuria in obese rats. Int. Urol. Nephrol..

[87] Bassiri-Tehrani B., Karanetz I., Bernik S.F., Dec W., Lehman J.C., Lerman O.Z. (2018). The Timing of Chemoprophylaxis in Autologous Microsurgical Breast Reconstruction. Plast. Reconstr. Surg..

[88] Mahabadi A.A., Massaro J.M., Rosito G.A., Levy D., Murabito J.M., Wolf P.A., O’Donnell C.J., Fox C.S., Hoffmann U. (2009). Association of pericardial fat, intrathoracic fat, and visceral abdominal fat with cardiovascular disease burden: The Framingham Heart Study. Eur. Heart J..

[89] Manno C., Campobasso N., Nardecchia A., Triggiani V., Zupo R., Gesualdo L., Silvestris F., De Pergola G. (2019). Relationship of para- and perirenal fat and epicardial fat with metabolic parameters in overweight and obese subjects. Eat. Weight. Disord..

[90] De Pergola G., Campobasso N., Nardecchia A., Triggiani V., Caccavo D., Gesualdo L., Silvestris F., Manno C. (2015). Para- and perirenal ultrasonographic fat thickness is associated with 24-hours mean diastolic blood pressure levels in overweight and obese subjects. BMC Cardiovasc. Disor.

[91] Wang W., Lv F.Y., Tu M., Guo X.L. (2024). Perirenal fat thickness contributes to the estimated 10-year risk of cardiovascular disease and atherosclerotic cardiovascular disease in type 2 diabetes mellitus. Front. Endocrinol..

[92] Lim S. (2014). Ectopic fat assessment focusing on cardiometabolic and renal risk. Endocrinol. Metab..

[93] Bragina A.E., Osadchiy K.K., Rodionova J.N., Bayutina D.A., Cherepanov G., Podzolkov V.I. (2022). Pararenal Fat and Renal Dysfunction in Patients without Significant Cardiovascular Disease. Am. J. Nephrol..

[94] Sun X., Han F., Miao W., Hou N., Cao Z., Zhang G. (2013). Sonographic evaluation of para- and perirenal fat thickness is an independent predictor of early kidney damage in obese patients. Int. Urol. Nephrol..

[95] Sun X., Yu Y., Han L. (2013). High FFA levels related to microalbuminuria and uncoupling of VEGF-NO axis in obese rats. Int. Urol. Nephrol..

[96] Kaneko K., Mitsuno R., Kojima D., Azegami T., Kosugi S., Nakamura T., Hashiguchi A., Yamada Y., Jinzaki M., Yamaguchi S. (2024). Renal sinus fat is associated with intrarenal hemodynamic abnormalities independent of visceral fat in patients with chronic kidney disease. Obes. Res. Clin. Pract..

[97] Mende C., Einhorn D. (2022). Fatty kidney disease: The importance of ectopic fat deposition and the potential value of imaging. J. Diabetes.

[98] Moriconi D., Mengozzi A., Duranti E., Cappelli F., Taddei S., Nannipieri M., Bruno R.M., Virdis A. (2023). The renal resistive index is associated with microvascular remodeling in patients with severe obesity. J. Hypertens..

[99] Jung M.H., Ihm S.H. (2023). Obesity-related hypertension and chronic kidney disease: From evaluation to management. Kidney Res. Clin. Pract..

[100] Hall J.E., do Carmo J.M., da Silva A.A., Wang Z., Hall M.E. (2019). Obesity, kidney dysfunction and hypertension: Mechanistic links. Nat. Rev. Nephrol..

[101] Pei K., Gui T., Li C., Zhang Q., Feng H., Li Y., Wu J., Gai Z. (2020). Recent Progress on Lipid Intake and Chronic Kidney Disease. Biomed. Res. Int..

[102] Wang Y.C., Feng Y., Lu C.Q., Ju S. (2018). Renal fat fraction and diffusion tensor imaging in patients with early-stage diabetic nephropathy. Eur. Radiol..

[103] Spurny M., Jiang Y., Sowah S.A., Nonnenmacher T., Schubel R., Kirsten R., Johnson T., von Stackelberg O., Ulrich C.M., Kaaks R. (2022). Changes in Kidney Fat upon Dietary-Induced Weight Loss. Nutrients.

[104] Lin T.Y., Liu J.S., Hung S.C. (2018). Obesity and risk of end-stage renal disease in patients with chronic kidney disease: A cohort study. Am. J. Clin. Nutr..

[105] Standring S. (2020). Gray’s Anatomy: The Anatomical Basis of Clinical Practice.

[106] Islam M.T., Chowdhury A.T., Siraj M.S., Md Abdullah A.Y., Mazumder T., Trask M., Talukder M.R., Rahman S.M. (2024). Anthropometric indices in predicting 10-year cardiovascular risk among males and females aged 40–74 years in south and southeast Asia: Analysis of 12 WHO STEPS survey data. Lancet Reg. Health Southeast Asia.

[107] Park M.J., Hwang S.Y., Kim N.H., Kim S.G., Choi K.M., Baik S.H., Yoo H.J. (2023). A Novel Anthropometric Parameter, Weight-Adjusted Waist Index Represents Sarcopenic Obesity in Newly Diagnosed Type 2 Diabetes Mellitus. J. Obes. Metab. Syndr..

[108] Liu J., Tse L.A., Liu Z., Rangarajan S., Hu B., Yin L., Leong D.P., Li W., China P.s.i. (2019). Predictive Values of Anthropometric Measurements for Cardiometabolic Risk Factors and Cardiovascular Diseases Among 44 048 Chinese. J. Am. Heart Assoc..

[109] Wei X., Min Y., Song G., Ye X., Liu L. (2024). Association between triglyceride-glucose related indices with the all-cause and cause-specific mortality among the population with metabolic syndrome. Cardiovasc. Diabetol..

[110] Lopez-Jaramillo P., Gomez-Arbelaez D., Martinez-Bello D., Abat M.E.M., Alhabib K.F., Avezum A., Barbarash O., Chifamba J., Diaz M.L., Gulec S. (2023). Association of the triglyceride glucose index as a measure of insulin resistance with mortality and cardiovascular disease in populations from five continents (PURE study): A prospective cohort study. Lancet Healthy Longev..

[111] Krakauer N.Y., Krakauer J.C. (2012). A new body shape index predicts mortality hazard independently of body mass index. PLoS ONE.

[112] Samouda H., Dutour A., Chaumoitre K., Panuel M., Dutour O., Dadoun F. (2013). VAT=TAAT-SAAT: Innovative anthropometric model to predict visceral adipose tissue without resort to CT-Scan or DXA. Obesity.

[113] Mazidi M., Kengne A.P., Katsiki N., Mikhailidis D.P., Banach M. (2018). Lipid accumulation product and triglycerides/glucose index are useful predictors of insulin resistance. J. Diabetes Complicat..

[114] Kobori H., Mori H., Masaki T., Nishiyama A. (2013). Angiotensin II blockade and renal protection. Curr. Pharm. Des..

[115] Ruilope L.M. (2005). Renin-angiotensin-aldosterone system blockade and renal protection: Angiotensin-converting enzyme inhibitors or angiotensin II receptor blockers?. Acta Diabetol..

[116] Engeli S., Schling P., Gorzelniak K., Boschmann M., Janke J., Ailhaud G., Teboul M., Massiera F., Sharma A.M. (2003). The adipose-tissue renin-angiotensin-aldosterone system: Role in the metabolic syndrome?. Int. J. Biochem. Cell Biol..

[117] Zhang R.B., Reisin E. (2000). Obesity-hypertension: The effects on cardiovascular and renal systems. Am. J. Hypertens..

[118] Sato A. (2019). Does the temporary decrease in the estimated glomerular filtration rate (eGFR) after initiation of mineralocorticoid receptor (MR) antagonist treatment lead to a long-term renal protective effect?. Hypertens. Res..

[119] Cheang W.S., Tian X.Y., Wong W.T., Lau C.W., Lee S.S., Chen Z.Y., Yao X., Wang N., Huang Y. (2014). Metformin protects endothelial function in diet-induced obese mice by inhibition of endoplasmic reticulum stress through 5′ adenosine monophosphate-activated protein kinase-peroxisome proliferator-activated receptor delta pathway. Arterioscler. Thromb. Vasc. Biol..

[120] Duca F.A., Cote C.D., Rasmussen B.A., Zadeh-Tahmasebi M., Rutter G.A., Filippi B.M., Lam T.K. (2015). Metformin activates a duodenal Ampk-dependent pathway to lower hepatic glucose production in rats. Nat. Med..

[121] Awal H.B., Nandula S.R., Domingues C.C., Dore F.J., Kundu N., Brichacek B., Fakhri M., Elzarki A., Ahmadi N., Safai S. (2020). Linagliptin, when compared to placebo, improves CD34+ve endothelial progenitor cells in type 2 diabetes subjects with chronic kidney disease taking metformin and/or insulin: A randomized controlled trial. Cardiovasc. Diabetol..

[122] Nakao M., Shimizu I., Katsuumi G., Yoshida Y., Suda M., Hayashi Y., Ikegami R., Hsiao Y.T., Okuda S., Soga T. (2021). Empagliflozin maintains capillarization and improves cardiac function in a murine model of left ventricular pressure overload. Sci. Rep..

[123] Zhou Y., Tai S., Zhang N., Fu L., Wang Y. (2023). Dapagliflozin prevents oxidative stress-induced endothelial dysfunction via sirtuin 1 activation. Biomed. Pharmacother..

[124] Lardaro A., Quarta L., Pagnotta S., Sodero G., Mariani S., Del Ben M., Desideri G., Ettorre E., Baratta F. (2024). Impact of Sodium Glucose Cotransporter 2 Inhibitors (SGLT2i) Therapy on Dementia and Cognitive Decline. Biomedicines.

[125] Pawlos A., Broncel M., Wozniak E., Gorzelak-Pabis P. (2021). Neuroprotective Effect of SGLT2 Inhibitors. Molecules.

[126] Lim D.M., Park K.Y., Hwang W.M., Kim J.Y., Kim B.J. (2017). Difference in protective effects of GIP and GLP-1 on endothelial cells according to cyclic adenosine monophosphate response. Exp. Ther. Med..

[127] Liu H., Hu Y., Simpson R.W., Dear A.E. (2008). Glucagon-like peptide-1 attenuates tumour necrosis factor-alpha-mediated induction of plasminogen [corrected] activator inhibitor-1 expression. J. Endocrinol..

[128] Sourris K.C., Ding Y., Maxwell S.S., Al-Sharea A., Kantharidis P., Mohan M., Rosado C.J., Penfold S.A., Haase C., Xu Y. (2024). Glucagon-like peptide-1 receptor signaling modifies the extent of diabetic kidney disease through dampening the receptor for advanced glycation end products-induced inflammation. Kidney Int..

[129] Gotoh K., Masaki T., Chiba S., Ando H., Fujiwara K., Shimasaki T., Tawara Y., Toyooka I., Shiraishi K., Mitsutomi K. (2013). Effects of hydrophilic statins on renal tubular lipid accumulation in diet-induced obese mice. Obes. Res. Clin. Pract..

[130] Valle Flores J.A., Farino Cortez J.E., Mayner Tresol G.A., Perozo Romero J., Blasco Carlos M., Nestares T. (2020). Oral supplementation with omega-3 fatty acids and inflammation markers in patients with chronic kidney disease in hemodialysis. Appl. Physiol. Nutr. Metab..

[131] Rund K.M., Peng S., Greite R., Claassen C., Nolte F., Oger C., Galano J.M., Balas L., Durand T., Chen R. (2020). Dietary omega-3 PUFA improved tubular function after ischemia induced acute kidney injury in mice but did not attenuate impairment of renal function. Prostaglandins Other Lipid Mediat..

[132] Bussolati B., Deregibus M.C., Fonsato V., Doublier S., Spatola T., Procida S., Di Carlo F., Camussi G. (2005). Statins prevent oxidized LDL-induced injury of glomerular podocytes by activating the phosphatidylinositol 3-kinase/AKT-signaling pathway. J. Am. Soc. Nephrol..

[133] Hong Y.A., Lim J.H., Kim M.Y., Kim T.W., Kim Y., Yang K.S., Park H.S., Choi S.R., Chung S., Kim H.W. (2014). Fenofibrate Improves Renal Lipotoxicity through Activation of AMPK-PGC-1α in Mice. PLoS ONE.

[134] Davis T.M., Ting R., Best J.D., Donoghoe M.W., Drury P.L., Sullivan D.R., Jenkins A.J., O’Connell R.L., Whiting M.J., Glasziou P.P. (2011). Effects of fenofibrate on renal function in patients with type 2 diabetes mellitus: The Fenofibrate Intervention and Event Lowering in Diabetes (FIELD) Study. Diabetologia.

[135] Ting R.D., Keech A.C., Drury P.L., Donoghoe M.W., Hedley J., Jenkins A.J., Davis T.M., Lehto S., Celermajer D., Simes R.J. (2012). Benefits and safety of long-term fenofibrate therapy in people with type 2 diabetes and renal impairment: The FIELD Study. Diabetes Care.

[136] Suzuki H., Watanabe Y., Kumagai H., Shuto H. (2013). Comparative efficacy and adverse effects of the addition of ezetimibe to statin versus statin titration in chronic kidney disease patients. Ther. Adv. Cardiovasc. Dis..

[137] Jung S.M., Kim C.T., Kang E.W., Kim K.H., Lee S., Oh H.J., Kim S.J., Kang D.H., Choi K.B., Ryu D.R. (2017). Dementia is a risk factor for major adverse cardiac and cerebrovascular events in elderly Korean patients initiating hemodialysis: A Korean national population-based study. BMC Nephrol..

[138] Haynes R., Lewis D., Emberson J., Reith C., Agodoa L., Cass A., Craig J.C., de Zeeuw D., Feldt-Rasmussen B., Fellstrom B. (2014). Effects of lowering LDL cholesterol on progression of kidney disease. J. Am. Soc. Nephrol..

[139] Itano S., Kadoya H., Satoh M., Nakamura T., Murase T., Sasaki T., Kanwar Y.S., Kashihara N. (2020). Non-purine selective xanthine oxidase inhibitor ameliorates glomerular endothelial injury in Ins(Akita) diabetic mice. Am. J. Physiol. Renal Physiol..

[140] Bae J., Hong N., Lee B.W., Kang E.S., Cha B.S., Lee Y.H. (2020). Comparison of Renal Effects of Ezetimibe-Statin Combination versus Statin Monotherapy: A Propensity-Score-Matched Analysis. J. Clin. Med..

[141] Byun J.H., Lebeau P.F., Platko K., Carlisle R.E., Faiyaz M., Chen J., MacDonald M.E., Makda Y., Yousof T., Lynn E.G. (2022). Inhibitory Antibodies against PCSK9 Reduce Surface CD36 and Mitigate Diet-Induced Renal Lipotoxicity. Kidney360.

[142] Wu D., Zhou Y., Pan Y., Li C., Wang Y., Chen F., Chen X., Yang S., Zhou Z., Liao Y. (2020). Vaccine Against PCSK9 Improved Renal Fibrosis by Regulating Fatty Acid beta-Oxidation. J. Am. Heart Assoc..

[143] Rangel Silvares R., Nunes Goulart da Silva Pereira E., Eduardo Ilaquita Flores E., Lino Rodrigues K., Ribeiro Silva A., Goncalves-de-Albuquerque C.F., Daliry A. (2019). High-fat diet-induced kidney alterations in rats with metabolic syndrome: Endothelial dysfunction and decreased antioxidant defense. Diabetes Metab. Syndr. Obes..

[144] Noce A., Di Lauro M., Di Daniele F., Pietroboni Zaitseva A., Marrone G., Borboni P., Di Daniele N. (2021). Natural Bioactive Compounds Useful in Clinical Management of Metabolic Syndrome. Nutrients.

[145] Tousson E., El-Sayed I.E.T., Elsharkawy H.N., Ahmed A.S. (2024). Ameliorating and Therapeutic Impact of Curcumin Nanoparticles Against Aluminum Oxide Nanoparticles Induced Kidney Toxicity, DNA Damage, Oxidative Stress, PCNA and TNFalpha Alteration in Male Rats. Environ. Toxicol..

[146] Ceja-Galicia Z.A., Garcia-Arroyo F.E., Aparicio-Trejo O.E., El-Hafidi M., Gonzaga-Sanchez G., Leon-Contreras J.C., Hernandez-Pando R., Guevara-Cruz M., Tovar A.R., Rojas-Morales P. (2022). Therapeutic Effect of Curcumin on 5/6Nx Hypertriglyceridemia: Association with the Improvement of Renal Mitochondrial beta-Oxidation and Lipid Metabolism in Kidney and Liver. Antioxidants.

[147] Feng Q., Yang Y., Qiao Y., Zheng Y., Yu X., Liu F., Wang H., Zheng B., Pan S., Ren K. (2023). Quercetin Ameliorates Diabetic Kidney Injury by Inhibiting Ferroptosis via Activating Nrf2/HO-1 Signaling Pathway. Am. J. Chin. Med..

[148] Kim S.R., Jiang K., Ogrodnik M., Chen X., Zhu X.Y., Lohmeier H., Ahmed L., Tang H., Tchkonia T., Hickson L.J. (2019). Increased renal cellular senescence in murine high-fat diet: Effect of the senolytic drug quercetin. Transl. Res..

[149] Liu Y.F., Wang H.H., Geng Y.H., Han L., Tu S.H., Wang H. (2023). Advances of berberine against metabolic syndrome-associated kidney disease: Regarding effect and mechanism. Front. Pharmacol..

[150] Zhang G., Lin X., Shao Y., Su C., Tao J., Liu X. (2020). Berberine reduces endothelial injury and arterial stiffness in spontaneously hypertensive rats. Clin. Exp. Hypertens..

[151] Rong Q., Han B., Li Y., Yin H., Li J., Hou Y. (2021). Berberine Reduces Lipid Accumulation by Promoting Fatty Acid Oxidation in Renal Tubular Epithelial Cells of the Diabetic Kidney. Front. Pharmacol..

